# Dual-Cross-Linked Alginate Hydrogels as a Strategy to Improve the Antifungal Properties of Posaconazole

**DOI:** 10.3390/pharmaceutics17081055

**Published:** 2025-08-14

**Authors:** Katarzyna Sosnowska, Marta Szekalska, Ewelina Piktel, Robert Bucki, Eliza Wolska, Iwona Misztalewska-Turkowicz, Karolina Halina Markiewicz, Agnieszka Zofia Wilczewska, Katarzyna Winnicka

**Affiliations:** 1 Department of Pharmaceutical Technology, Medical University of Białystok, Mickiewicza 2C, 15-222 Białystok, Poland; marta.szekalska@umb.edu.pl (M.S.); katarzyna.winnicka@umb.edu.pl (K.W.); 2 Independent Laboratory of Nanomedicine, Medical University of Białystok, 15-222 Białystok, Poland; ewelina.piktel@umb.edu.pl; 3 Department of Medical Microbiology and Nanobiomedical Engineering, Medical University of Białystok, 15-222 Białystok, Poland; buckirobert@gmail.com; 4 Department of Pharmaceutical Technology, Medical University of Gdańsk, Hallera 107, 80-416 Gdańsk, Poland; eliw@gumed.edu.pl; 5 Department of Polymers and Organic Synthesis, Faculty of Chemistry, University of Białystok, Ciołkowskiego 1K, 15-245 Białystok, Poland; i.misztalewska@uwb.edu.pl (I.M.-T.); k.markiewicz@uwb.edu.pl (K.H.M.); agawilczuwb@gmail.com (A.Z.W.)

**Keywords:** sodium alginate, ε-poly-L-lysine, hydrogels, cross-linking, posaconazole

## Abstract

**Background/Objectives**: Despite the continuous development of medicine, the treatment of dermatological fungal infections is difficult due to their chronic nature, recurrence, and resistance of some pathogens to standard therapies. In order to improve the effectiveness of treatment, not only are new active substances with antifungal activity synthesized, but new, unconventional carriers are also developed for substances already used. **Methods**: Therefore, the focus of this research was to evaluate the possibility of using a combination of two cross-linking techniques for sodium alginate ionic cross-linking with Zn^2+^ ions and electrostatic interaction with ε-poly-L-lysine. The pharmaceutical properties, antifungal activity against *Candida* strains, and compatibility with human fibroblasts of the designed hydrogels were assessed. **Results**: It was shown that the double cross-linking process increased the viscosity of the developed hydrogels, improved bioadhesive properties to hairless mice skin, and provided an extended release profile of the active substance. In addition, obtained formulations were characterized by improved antifungal effect against *C. albicans*, *C. krusei*, and *C. parapsilosis*. Prepared hydrogels expressed biocompatibility with human fibroblasts. **Conclusions**: Dual-cross-linked alginate hydrogels are a promising dermatological formulation that might improve the efficacy of posaconazole in the treatment of antifungal infections.

## 1. Introduction

Hydrogels are specific, three-dimensional hydrophilic networks of polymers, which, despite the high water compactness, show a stable structure. The capacity of water absorption and stability of the hydrogel network are controlled by networking, which includes creating bonds between polymer chains. Hydrogels are crossed, partly permanent drug carriers with favourable plasticity, bio- and mucoadhesiveness. Due to the presence of large amounts of water in their structure, they are characterized by adequate plasticity, good washability, and similarity to natural tissues, therefore finding applications in medicine and pharmacy. In addition, the presence of water in hydrogels provides cooling, moisturizing, and pain relief properties, as well as supporting wound healing processes, which is beneficial in many dermatological diseases [1,2].

Organic polymers (e.g., cellulose derivatives, acrylic acid derivatives, or polysaccharides) are mainly used to obtain hydrogels. The group of polysaccharide polymers includes, among others, sodium alginate–a polymer of natural origin, characterized by biocompatibility and biodegradability [1]. Sodium alginate (ALG) is the sodium salt of alginic acid, composed of β-D-mannuronic acid and α-L-guluronic acid monomers connected by (1–4) glycosidic bonds. It possesses the capacity for swelling, forming a gel, and muco- and bioadhesion, which support its application in formulating various types of drug carriers, including hydrogels. Improvements in the physical and mechanical properties of the polymer can be achieved by modifications involving cross-linking with various agents or combining it with other polymers [3,4]. ALG is distinguished by its ability to form hydrogels through ionic cross-linking with divalent or trivalent metal cations in a process commonly known as the “egg-box” model. This process involves reaction of the guluronic acid blocks of the ALG chains with multivalent cations, resulting in the formation of a three-dimensional gel network. Calcium chloride (CaCl_2_) is the most widely employed source of divalent cations due to its high solubility and effective gelation capability. ALG cross-linking with calcium ions exhibits certain limitations. Among other things, the process occurs quickly and in an uncontrolled manner, and the resulting gels do not always show favourable mechanical properties [5]. Recent advancements in ALG gelation strategies have explored the utilization of alternative metal ions, with the aim of modulating the structural and functional properties of the resulting hydrogels. Notably, zinc ions (Zn^2+^) have garnered interest due to their enhanced interaction with the carboxyl groups of alginate, potentially leading to a more extensive and stable cross-linked network. The superior coordination ability of Zn^2+^ may facilitate the formation of denser gel matrices compared to those obtained using Ca^2+^. In addition to their gelation efficacy, zinc ions possess well-documented antifungal properties, making them particularly advantageous in biomedical applications. Specifically, the incorporation of Zn^2+^ in alginate-based hydrogels has potential utility in the formulation of dermatological preparations for the treatment of cutaneous mycoses, where both mechanical integrity and antimicrobial activity are desired [6]. Another way to modify the ALG structure is to combine it with cationic polymers, creating polyelectrolyte complexes (PEC). These are association complexes formed as a result of electrostatic interactions between molecules with the opposite charge (e.g., polymer–polymer, polymer–drug and polymer–drug–polymer) [7]. The ALG anionic nature may promote the PEC formation with ε-poly-L-lysine (PLL), a positively charged cationic peptide (a natural homopolypeptide consisting of about 25–30 L-lysine residues) [8,9]. The Food and Drug Administration (FDA) classifies PLL as a safe substance (Generally Regarded as Safe, GRAS) [10]. Due to many valuable properties such as non-toxicity, biodegradability, and bacteriostatic and antifungal activity, PLL is widely used in medicine and pharmacy [10,11]. Due to their characteristics, PLL and ALG can be applied in a single product. PLL and ALG were employed to prepare hydrogel films with strong antibacterial properties in a broad pH range. These films showed very good cell compatibility, suggesting that they could be used as novel wound healing product [12]. A polyelectrolyte complex of PLL and ALG was also applied to achieve a material exhibiting mechanical features similar to natural tissues and with the ability to self-heal [13]. Furthermore, ALG itself has proved to be a good carrier for antifungal drugs, mostly azoles and polyenes. In recent years, different alginate delivery systems for antimycotic substances have been examined, for example, films, hydrogels, tablets, beads, and micro- or nanoparticles [14,15,16]. In our previous research comparing ALG hydrogel and cryogel, we showed that the methods of obtaining alginate hydrogels affects the rate of release of the antifungal substance. Diffusion of POS from cryogels was prolonged. It was also observed that cryogel formulation inhibited the growth of *C. parapsilosis* more strongly than hydrogel [15].

Posaconazole (POS) is a broad-spectrum, relatively new triazole antifungal active substance with activity against, e.g., *Candida* spp., *Cryptococcus neoformans*, *Cryptococcus gattii*, *Aspergillus* spp., *Scedosporium apiospermum*, *Fusarium oxysporum*. It is also active against dermatophytes, including *Trichophyton tonsurans* and *Trichophyton rubrum* [17]. POS is applied primarily for the prevention and treatment of invasive fungal infections, particularly in immunocompromised patients. It exerts its antifungal activity by inhibiting the enzyme lanosterol 14α-demethylase, thereby disrupting ergosterol synthesis and compromising fungal cell membrane integrity [18,19]. Although POS’s antifungal efficacy against yeast and dermatophytes is well established [18,19], no topical formulations containing POS are currently commercially available [20]. Therefore, investigations were conducted to explore the potential of utilizing the compound in formulations intended for topical dermal application.

There are literature reports indicating that utilization of the double cross-linking method may constitute an effective strategy to improve mechanical, rheological and bioadhesive properties [21,22,23]. Therefore, the aim of this work was to assess the possibility of employing the application of a combination of two cross-linking techniques–ionic cross-linking with Zn^2+^ ions and ALG electrostatic interaction with PLL. In the next stage, the impact of the addition of cross-linking agents on the pharmaceutical properties of the hydrogels was also assessed. Additionally, the antifungal activity of the designed hydrogels against *Candida* strains *C. albicans*, *C. krusei* and *C. parapsilosis* and compatibility with human fibroblasts was analyzed.

## 2. Materials and Methods

Posaconazole (POS) and ε-poly-L-lysine (PLL) were acquired from Kerui Biotechnology Co., Ltd. (Xi’an, China). Sodium alginate (ALG) from *Macrocystis pyrifera* with viscosity 2415 mPa·s for 1% solution at 25 °C (containing 61% mannuronic acid (M) and 39% guluronic acid (G) (M/G ratio of 1.56) characterized by molecular weight 3.5 × 10^5^ Da) and zinc acetate dihydrate (Zn(OAc)_2_·2H_2_O) were procured from Sigma Aldrich St. Louis, MO, USA. Natural cellulose membrane Cuprophan^®^, characterized by 10,000 Da molecular weight cut-off, was purchased from Medicell (London, UK). Hairless mouse (Cby.Cg-Foxn1nu/cmdb) skin from the dorsal area was received from the Experimental Medicine Center of the Medical University of Białystok (approval of the Local Ethical Committee for Experiments on Animals was not required). The stock cultures of *Candida albicans* ATCC^®^ 10231, *Candida krusei* ATCC^®^ 6528, *Candida parapsilosis* ATCC^®^ 22019 and Sabouraud dextrose agar were acquired from Biomaxima (Lublin, Poland). Additionally, BD Difco™ Sabouraud Dextrose Broth and BD DIFCO™ Sabouraud Dextrose Agar media were purchased from Argenta (Poznań, Poland). All other reagents were of analytical grade or HPLC grade.

### 2.1. Cell Culture Materials

Human skin fibroblast cell line (CCD-1070Sk (CRL-2091™), Eagle’s Minimum Essential Medium (EMEM) cell culture medium, and fetal bovine serum (FBS) were purchased from American Type Culture Collection (ATCC, Manassas, VA, USA). Phosphate-buffered saline solution (PBS) without calcium and magnesium was from PAN Biotech (Aidenbach, Germany), while 10X Trypsin-EDTA solution and Penicillin-Streptomycin solution were from Sigma Aldrich (St. Louis, MO, USA). The final cell culture medium was prepared by supplementing EMEM with FBS (to a final concentration of 10%) and antibiotics (to final penicillin and streptomycin concentrations of 50 U/mL and 50 µg/mL, respectively). In order to observe the cytoskeleton and nuclei, we applied formaline 36–38% (Chempur, Piekary Śląskie, Poland) and Triton X-100 (Sigma Aldrich, St. Louis, Missouri, USA) for cell stabilization. For precise evaluation purposes, fluorescent FITC-labelled phalloidin (Thermo Fisher Scientific, Waltham, MA, USA), bisBenzimide H (Thermo Fisher Scientific, Waltham, MA, USA), and non-fluorescent resazurin sodium salt (Sigma Aldrich, St. Louis, MO, USA) dyes were utilized.

### 2.2. ALG/PLL Polyelectrolyte Complex Development and Evaluation

Polymer mixtures containing 2% ALG solution and various concentrations (0.1%, 0.5% and 1%) of PLL were formulated by applying a RZR 2020 mechanical stirrer (Heidolph Instruments, Schabach, Germany) [13]. After 24 h, a rotational viscometer (RVDV-III Ultra, Brookfield Engineering Laboratories, Middleboro, MA, USA) fitted with a CPA52Z cone (plate diameter 24 mm, cone angle 3°) was utilized for viscosity measurement at shear rate of 2.00 s^−1^ and constant temperature of 22 ± 1 °C. Turbidity assessment was performed by using a Hach Model 2100 N IS^®^ Laboratory Turbidimeter (Hach Company, Loveland, CO, USA) and stated in nephelometric turbidity unit (NTU).

### 2.3. Dual-Cross-Linked Hydrogels Formation

Firstly, the 2% (*w*/*w*) ALG solution was prepared and adjusted to pH 5.0 with 0.1 M HCl pH 1.2. Then, 5 mL 0.1% (*w*/*w*) PLL solution (pH 5.0) was added dropwise into the ALG solution [13]. Next, 5 mL of zinc acetate dihydrate (Zn(OAc)_2_·2H_2_O) in different concentrations (1–5%) was dripped into the ALG/PLL solution under constant stirring to acquire homogenous dual-cross-linked hydrogels. After 24 h, a rotational viscometer (RVDV-III Ultra, Brookfield Engineering Laboratories, Middleboro, MA, USA) fitted with a CPA52Z cone (plate diameter 24 mm, cone angle 3°) was utilized for viscosity measurement at a shear rate of 2.00 s^−1^ and constant temperature of 22 ± 1 °C. To prepare drug-loaded hydrogels, POS at 1.0% *w*/*w* concentration was evenly distributed in gel carriers (Table 1). Non-cross-linked ALG hydrogel and hydrogels without active substance (formulation P1–P3) were used as a control. Then, 24 h after preparation, hydrogels were subjected to pH evaluation by the pH meter Orion 3 Star (Thermo Scientific, Waltham, MA, USA).

### 2.4. Dual-Cross-Linked Hydrogel Assessment

#### 2.4.1. Solid-State Characterization

For Scanning Electron Microscope (SEM) evaluation, samples of the dual-cross-linked hydrogels (5 g) were placed in plastic vials and lyophilized by using a freeze-dryer (Alpha 2–4 LSC Basic, Martin Christ, Osterode am Harz, Germany) at −20 °C for 24 h [24]. Sputter-coated samples of lyophilizates with a 2 nm thick gold layer were imaged by microscope Phenom Pro G5 (Phenom World, Eindhoven, the Netherlands). Samples of hydrogels (1 mg) were observed using an optical microscope Motic BA 400 (Moticon, Wetzlar, Germany) with total magnifications of 100×, 400× and 1000×. POS particles were observed and measured in at least three different areas of observation.

#### 2.4.2. POS Content and Particle Analysis

The POS concentration was assessed following the extraction of 1 g hydrogel formulation in 2 mL of water, stirred for 1 h at 75 rpm in a heated water bath (37 ± 1 °C). Subsequently, 8 mL of methanol was introduced and mixed for 24 h. The mixture samples were analyzed at 260 nm utilizing High-Performance Liquid Chromatography (HPLC) as outlined in Section High-Performance Liquid Chromatography (HPLC) Method. To assess POS particle size hydrogels were observed using an optical microscope Motic BA 400 (Moticon, Wetzlar, Germany) under magnification 100×. 1 mg of each hydrogel formulation was placed on a glass slide and POS particles were measured in at least three different areas of observation [25].

##### High-Performance Liquid Chromatography (HPLC) Method

The POS concentration was assessed using the High-Performance Liquid Chromatography (HPLC) technique with an Agilent Technologies 1200 system (Agilent, Waldbronn, Germany) equipped with a Poroshell^®^ 120 EC-C18 2.7 μM ODS 4.6 × 150 mm, 2.7 μm column (Agilent, Waldbronn, Germany). The mobile phase was acetonitrile, methanol, and water (60:20:20, *v*/*v*) and flow rate was 0.5 mL/min. POS UV detection was performed at a wavelength of 260 nm and retention time was recorded at 5.3 min [26]. The standard calibration curve exhibited linearity within the range of 1–100 μg/mL, with a correlation coefficient (R^2^) of 0.998.

#### 2.4.3. Viscosity and Rheological Properties Analysis

The developed formulations were analyzed using a Brookfield rotational viscometer (RVDV-III Ultra, Brookfield Engineering Laboratories, Middleboro, MA, USA) fitted with a CPA52Z cone (plate diameter 24 mm, cone angle 3°) at a temperature of 22 ± 1 °C. The viscosity of prepared formulations was evaluated at shear rate 6.00 s^−1^. The recorded findings related to rheological behaviour were depicted as flow curves showing the relationship between viscosity and shear rate (2.00–10.00 s^−1^).

#### 2.4.4. Texture Analysis and Bioadhesiveness

Texture analysis and bioadhesiveness were evaluated using a TA.XT Plus texture analyzer (Stable Micro System, Godalming, UK). Firmness, consistency and cohesiveness were determined with applied disc (of 10 mm diameter), which was compressed at a rate of 1 mm/s for a depth of 5 mm into the evaluated samples (30 g) and then retracted. Bioadhesiveness was evaluated with A/MUC equipment and depicted as the detachment force (F_max_) and the work of adhesion (W_ad_). To mimic in vivo requirements the hairless mice skin was utilized. Pretest speed 0.5 mm/s, test speed 0.1 m/s, contact time 60 s, post-test speed 0.1 mm/s and applied force 0.5 N were selected in preliminary experiments. Data compilation and investigation were conducted with the Texture Exponent 32 software, version 5.0.

#### 2.4.5. In Vitro POS Release

The in vitro POS release was assessed using the USP dissolution apparatus 2 (Agilent 708-DS, Agilent Technologies, Cary, NC, USA) with mini vessels (250 mL) and mini paddles. The enhancer cell with a surface area of 3.80 cm^2^, consisted of a Teflon load ring, a cap, a natural cellulose membrane and a drug reservoir. Each dual-cross-linked hydrogel sample (2 g) was placed in the enhancer cell, which was then immersed in the dissolution vessel containing 100 mL of the release medium (water with the addition of 2% Tween 80 to receive the sink conditions). Tests were performed at 32 ± 1 °C corresponding to the skin surface temperature and with a speed of 75 rpm of mini paddles. Samples of 1 mL were collected at different time intervals (1, 2, and 3 h) and investigated at 260 nm using High-Performance Liquid Chromatography (HPLC), as described in Section High-Performance Liquid Chromatography (HPLC) Method. To explain the mechanism of POS release, data collected from POS release analysis were explored according to zero-order kinetics, first-order kinetics, the Higuchi model, and the Korsmeyer–Peppas equation [27].

#### 2.4.6. Thermal Analysis Performance and Attenuated Total Reflectance–Fourier Transform Infrared Spectroscopy (ATR–FTIR)

Thermal evaluation of the pure compounds (ALG, PLL, Zn(OAc)_2_·2H_2_O and POS) and lyophilized samples of dual-cross-linked hydrogels, controls, and POS-loaded formulations was conducted utilizing thermogravimetric (TGA) and differential scanning calorimetry analysis (DSC). TGA analyses were carried out in an inert gas atmosphere (argon flow 40 mL·min^−1^) over a temperature range of 50 to 900 °C, with a heating rate of 10 °C·min^−1^. DSC analysis, in turn, was performed under an inert gas flow of 200 mL·min^−1^, over a temperature range of 50 to 300 °C, with a heating rate of 10 °C·min^−1^. All ATR-FTIR (Attenuated Total Reflectance–Fourier Transform Infrared Spectroscopy) spectra were recorded using a Thermo Scientific Nicolet 6700 FTIR spectrophotometer (Waltham, MA, USA) equipped with an ATR accessory. Spectra were rationed against the background spectra and collected in the wavenumber range 4000 to 500 cm^−1^ by co-adding 32 scans with a resolution of 4 cm^−1^.

#### 2.4.7. Antifungal Activity

##### Agar Diffusion Method

Antifungal activity analysis of the designed hydrogel formulations were conducted according to the Clinical and Laboratory Standards Institute (CLSI) [28]. For tests, *Candida* cells of *C. albicans* ATCC^®^ 10231, *C. krusei* ATCC^®^ 6528 and *C. parapsilosis* ATCC^®^ 22019 were used. The optical density of the inoculum was examined by a suspension turbidity detector (Densitometer DEN-1B, Biosan, Riga, Latvia) and was found to be 0.5 in McFarland (5 × 10^6^ CFU/mL) in the sterile 0.9% NaCl. In agar plates, wells with a diameter of 5 mm were filled with prepared hydrogels formulations (100 mg). POS solution in DMSO (50 μL), pure substances ALG, PLL, and Zn^2+^ were applied as controls. After incubation at 37 ± 0.1 °C for 24 and 48 h, the growth inhibition zones were determined using a calliper (Mitutoyo, Kawasaki, Japan) with an accuracy of 0.1 mm.

##### Activity of Tested Formulations Against Planktonic and Biofilm Forms of Candida Fungi

To assess the antifungal potential of tested formulations, a colony-counting assay as well as resazurin-based biofilm viability assays were performed. In the first experimental setting, *Candida* strains were grown in Saboraud Broth to mid-log phase at 37 °C and adjusted to 10^5^ CFU/mL with sterile phosphate-buffered saline (PBS). Then, 100 µL of the prepared fungal suspensions were added to 100 µL of developed formulations, obtaining final concentrations of formulations of 10, 20, 33.7 or 50%. Upon incubation at 37 °C for 1 h, the plates were transferred to ice, formulation-inoculum mixtures were diluted 10- to 1000-fold in PBS, and 10 μL aliquots were spotted on Saboraud agar plates. CFUs were determined upon overnight culture at 37 °C and expressed as log (CFU/mL). To investigate the impact of the tested formulations on the biofilm formation ability, tested *Candida* strains were grown in Saboraud Broth to mid-log phase at 37 °C, adjusted to the optical density (OD) of ~0.1, and exposed in 96-well round bottom plates to tested formulations diluted to 1:10. Upon incubation at 37 °C for 24 h, the planktonic cells were removed and biofilm attached to the surface of the plates was washed twice with sterile PBS. Then, 100 µL of resazurin sodium salt solution (200 µg/mL) was added and after 1 h of incubation at 37 °C, the fluorescence signal was measured at excitation/emission wavelengths of 520/590 nm by applying microplate reader Varioskan LUX (Thermo Fisher Scientific, Waltham, MA, USA). Further, 1% POS solution in DMSO and PBS were applied as controls.

#### 2.4.8. Assessment of Biocompatibility of Tested Formulations Against Human Skin Fibroblasts

To investigate the biocompatibility of tested formulations against human skin fibroblasts, a cell culture model was employed. CCD-1070Sk (CRL-2091™) was maintained using Eagle’s Minimum Essential Medium (EMEM) supplemented with 10% of fetal bovine serum (FBS), glutamine (2 mM/L), penicillin (50 U/mL), and streptomycin (50 µg/mL) and maintained at 37 °C in an 5% CO_2_ atmosphere. Prior the experiment, cells were seeded in 6-well cell culture plates at a density of 5 × 10^5^ cells/well in 2 mL of medium. When nearly 100% confluence was obtained, 50 µL of undiluted formulation was applied to the centre of the well and then spread evenly over the surface of the well using a 30 mm coverslip. Then, 2 mL of cell culture medium was added before 4 or 24 h incubation at 37 °C. For the control, only untreated cells coverslips without formulation were applied. When analyzing the impact of POS on the cellular viability, a 1% suspension of POS in EMEM was used. At the indicated time points, the coverslips were removed, the cells were washed twice with sterile PBS to remove any residual of formulations, and they were fixed with 4% paraformaldehyde for 15 min at room temperature prior to permeabilization with 0.1% Triton X-100 and staining for cytoskeleton (using FITC-labelled phalloidin at 1 U/mL) and nuclei (using Hoechst at 1 µg/mL). The morphology of cells and the number of nuclei were examined via fluorescence microscopy using a fluorescence microscope with a live-imaging Leica DMi8 imaging system (Leica DMi8, Wetzlar, Germany).

### 2.5. Statistical Analysis

The received data were analyzed by using Statistica 12.0 software (StatSoft, Tulsa, OK, USA). Quantified variables were expressed as the mean and standard deviation. One-way analysis of variance (ANOVA) and the Kruskal–Wallis test were used as statistical tests. Differences between groups were determined to be significant at *p* < 0.05.

## 3. Results

ALG indicates distinctive gelling characteristics due to both chemical and/or physical cross-linking features. The most extensively used process for synthesizing alginate gels is the so-called “egg-box” cross-linking. This technique, also referred to as ionic gelation, takes place when divalent or trivalent cations interact with the guluronate segments of alginate chains, prompting the formation of a stable and robust three-dimensional gel network [4]. Among the most broadly implemented ALG cross-linking technique is Ca^2+^ ion utilization [3]. Nevertheless, their usage is associated with specific drawbacks, such as extremely accelerated and unstable polymer gelation. Consequently, alternative cross-linking agents are being explored. Zn^2+^ ions have been reported to exhibit stronger interactions with ALG, leading to more expansive cross-linking and a sustained active substance release profile versus Ca^2+^ ions. Furthermore, Zn^2+^ ions possess well-documented antifungal properties. Moreover, ALG is also capable of forming hydrogel matrices through the creation of polyelectrolyte complexes [5]. This phenomenon entails the interaction between the negatively charged carboxyl groups of alginate and positively charged polymers, such as chitosan, poly-L-lysine (PLL), or gelatin [4]. These polyelectrolyte complexes often demonstrate improved mechanical properties, excellent biocompatibility, and regulated release profiles, making them highly beneficial for use in drug delivery, wound repair, and various other biomedical applications [7]. The anionic carboxyl groups of ALG can engage in electrostatic interactions with the cationic groups present in the PLL molecule. Moreover, PLL, owing to its various beneficial attributes—such as non-toxicity, biodegradability, and bacteriostatic as well as antifungal activities—can further augment the antifungal efficacy of the resulting hydrogels [11].

**Figure 1:**
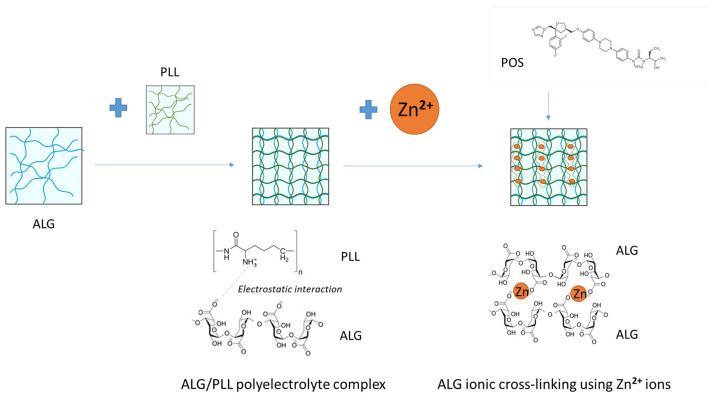
Design of ALG hydrogel dual-cross-linking by PLL and Zn^2+^ ions with POS.

This is supported by existing reports highlighting the impact of the dual-cross-linking process on hydrogel mechanical strength, mucoadhesive properties, and antifungal efficacy [21]. Therefore, a trial on double-cross-linking ALG using both PLL and Zn^2+^ ions was performed. In addition, the influence of the applied process on the properties of the designed hydrogels was assessed (Figure 1).

### 3.1. Turbidity and Viscosity Characteristics

**Figure 2:**
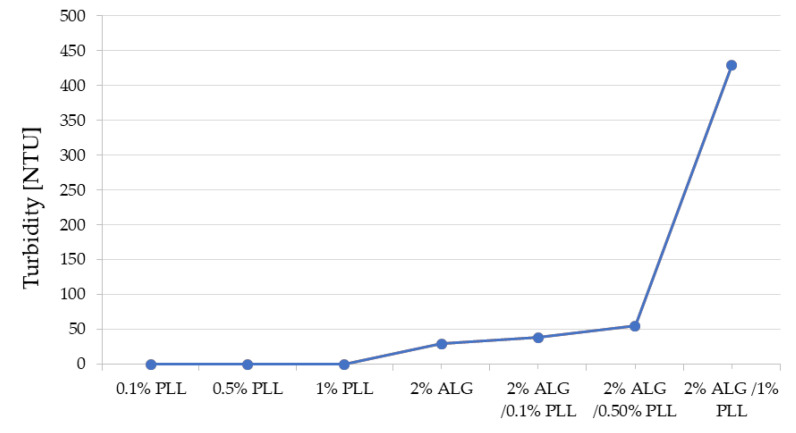
Turbidity values of 0.1%, 0.5%, 1% PLL, 2% ALG solutions and ALG/PLL PECs mixtures with regard to diverse polyanion/polycation proportions.

Based on previous experiments, ALG at a concentration of 2% was selected for testing as a formulation with optimal viscosity [15]. Data acquired from the turbidity assays indicated that the ALG/PLL complexes displayed higher turbidity levels than those observed for the separate polymer solutions (Figure 2). This observation substantiates the formation of ALG/PLL polyelectrolyte complexes (PECs) as a consequence of electrostatic attraction between the negatively charged carboxyl groups of ALG and the positively charged amine groups of PLL. Additionally, the turbidity of the ALG/PLL complexes was found to increase proportionally with rising PLL concentrations—higher PLL content promoted the generation of a greater number of PECs.

**Figure 3:**
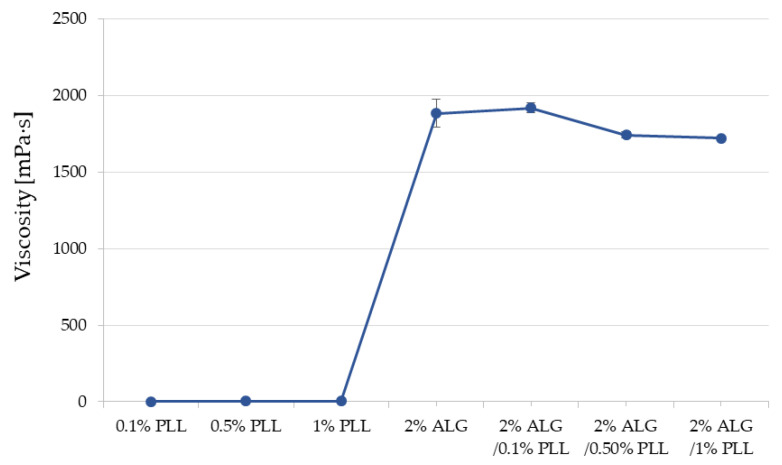
Viscosity values of 0.1%, 0.5%, 1% PLL, 2% ALG solutions, ALG/PLL PECs mixtures at multiple polyanion/polycation proportions (mean ± SD, *n* = 3).

The viscosity measurements of the 0.1%, 0.5%, 1% PLL, 2% ALG and ALG/PLL PECs mixtures concerning varied polyanion/polycation ratios are revealed in Figure 3. It was noted that both the ALG and 0.1% PLL solutions exhibited greater viscosity compared to the individual polymer solutions. Furthermore, the viscosity values progressively decreased with the elevation of PLL concentration.

**Figure 4:**
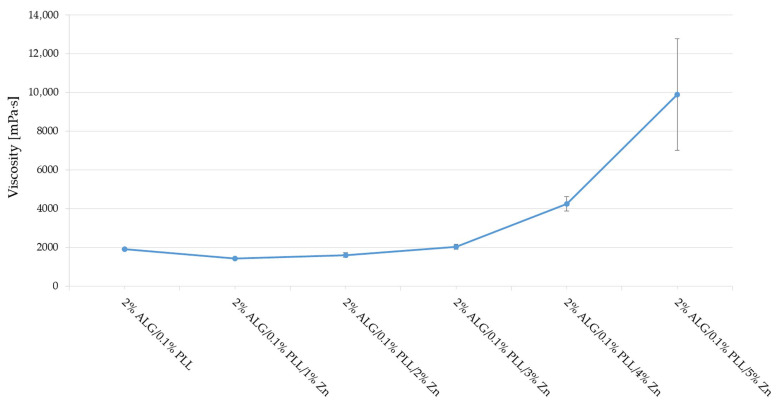
Viscosity values of 2% ALG/0.1% PLL PEC mixtures cross-linked with different concentrations of Zn^2+^ ions (mean ± SD, *n* = 3).

The cross-linking of ALG/PLL hydrogels utilizing different concentrations of Zn^2+^ was also evaluated (Figure 4). It was remarked that when 1% and 2% Zn^2+^ ions solutions were applied, the viscosity of the double-cross-linked hydrogels decreased. This suggests that the amount of Zn^2+^ ions was insufficient to form a stable hydrogel structure. In contrast, a 5% Zn^2+^ ion solution resulted in the formation of an inhomogeneous structure with high viscosity. The obtained results expressed that hydrogel consisting of 2% ALG cross-linked with 0.1 PLL and 4% Zn^2+^ ion solution were characterized by the optimal values of viscosity, turbidity, and clarity, and this formulation was subjected to further testing.

### 3.2. Assessment of ALG Dual-Cross-Linked Hydrogel Properties

Hydrogels constitute one of the forms of pharmaceutical preparations applied to the skin, mucous membranes, and wounds, and it may also be introduced into internal body cavities. They offer a practical alternative to lipophilic and absorptive bases, such as ointments and creams, owing to several application-related advantages. These benefits include, among others, the absence of a greasy layer on the skin post-application, ease of removal with water, and, most notably, the potential to exhibit mucoadhesive properties [29].

**Figure 5:**
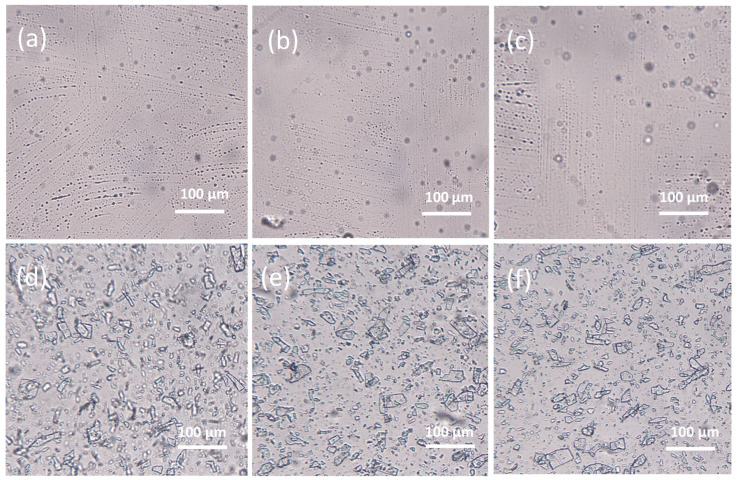
The representative SEM images of non-cross-linked and cross-linked hydrogel base (**a**) P1, (**b**) P2, (**c**) P3, and non-cross-linked and cross-linked hydrogel base containing POS (**d**) F1, (**e**) F2, (**f**) F3 under magnification ×200.

The generated dual-cross-linked hydrogel formulations were characterized by homogeneity and lack of phase separation. The characteristics of the formulations containing POS are featured in Table 2. The appropriate particle size in the case of a hydrogel suspension is relevant in the process of application, release, and action of the active substance [30]. It was noted that active the substance was evenly dispersed throughout the hydrogel base and its mean diameter was in the range from 22.87 ± 8.13 μm to 25.44 ± 9.46 μm (Table 2, Figure 5). The assessment of the active ingredient concentration revealed that the average POS content was within the acceptable USP pharmacopoeia range (90–110% of the labelled amount) [31], suggesting that the drug particles were evenly distributed (Table 2).

The pH values of the designed hydrogel formulations ranged from 5.04 ± 0.04 to 6.20 ± 0.01 (Table 2). The recorded results confirm the compatibility of the obtained formulations with the skin surface and the lack of risk of irritation—the physiological pH of the skin surface is moderately acidic and is in the range of pH 4.1–5.8 [32]. It was observed that the pH of the developed formulations insignificantly increased with the presence of cross-linking agents and POS compared to the hydrogel base.

The data received from the viscosity analysis of all developed formulations predictably showed that the cross-linking process contributed to an increase in this parameter (Table 2). It was demonstrated that the double cross-linking process significantly increased the viscosity of the obtained formulations, both those containing the drug substance and those without POS. Additionally, the inclusion of the drug substance was found to influence a decrease in viscosity (Table 2).

**Figure 6:**
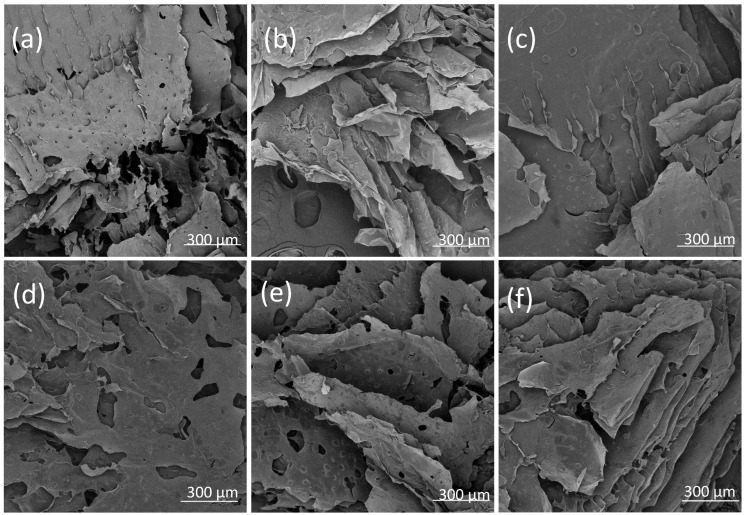
The representative microscopic images of prepared non-cross-linked and cross-linked hydrogel base (**a**) P1, (**b**) P2, (**c**) P3, and non-cross-linked and cross-linked hydrogel base containing POS (**d**) F1, (**e**) F2, (**f**) F3 under magnification ×100.

The morphology of the hydrogels in the placebo formulations and with the POS addition was examined using an optical microscope (Figure 5) and Scanning Electron Microscope (SEM) after the lyophilisation process (Figure 6). It was observed that the non-crosslinked formulation exhibited a porous structure. In contrast, double cross-linking with zinc ions resulted in hydrogels with a more robust and compact network. This relationship was also confirmed for formulations containing POS. Additionally, crystals of the active substance were visible in the samples. A homogenous and compact structure of alginate–norbornene hydrogel cross-linked by disulfide-tetrazine, as well as alginate–furfuryl amine hydrogel cross-linked by disulfide-maleimide, was also identified under SEM analysis by Siboro et al. [33].

### 3.3. Flow Curves and Textural Properties

**Figure 7:**
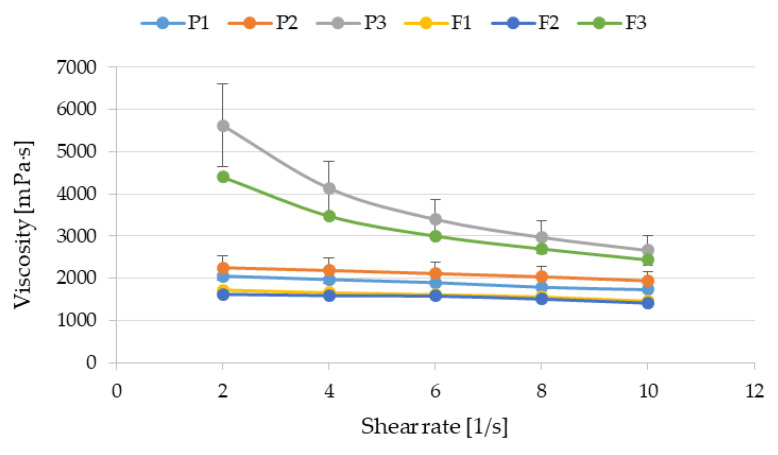
Viscosity plots versus shear rate of the dual-cross-linked hydrogel bases (formulations P1–P3) and hydrogels containing POS (formulations F1–F3) measured at 22 ± 1 °C (mean ± SD, *n* = 3).

Rheological and textural studies are used to estimate the structure of the dosage forms including hydrogels. One of the methods of assessing rheological properties is to generate/create flow curves, i.e., examining the dependence of the formulation viscosity on the shear rate. A decrease in viscosity with increasing shear rate is characteristic of non-Newtonian fluids, which are called shear-thinning fluids. Understanding these properties is useful when establishing the procedure for obtaining the preparations, mixing, choosing of the type of the packaging or method of application [34]. During our study, it was shown that all the examined formulations belonged to shear-thinning fluids (Figure 7). However, the greatest decline in the viscosity with the growing shear rate was noticed in P3 (without POS) and F3 (containing POS) preparations drawn up using cross-linking. 

This shear-thinning trend was also observed in many experiments irrespective of whether ALG hydrogels were prepared by addition of the cross-linking agents or other modification of the ALG structure [35,36,37,38]. It should be noted that higher viscosity of formulation F3 allows the above preparation to remain on the skin for longer time, thus providing a better therapeutic effect and higher antifungal activity. In addition, the increase in viscosity also contributes to prolonged release of the active substance.

**Figure 8:**
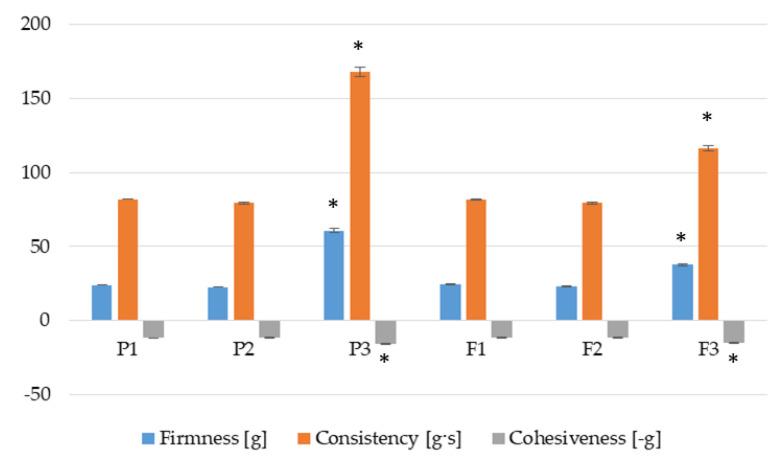
Textural properties of dual-cross-linked hydrogels (formulations P1–P3) and hydrogels containing POS (formulations F1–F3) (mean ± SD, *n* = 3, * significant statistical difference (*p* < 0.05) versus correspondent value of P1).

Textural evaluation involves measuring the product’s force response. The outcome of this study depends mainly on the structure of the tested formulations, whereas the structure results from the interactions between components. The firmness, consistency and cohesiveness of prepared hydrogels were analyzed. The firmness [g] and consistency [g·s] were indicated when the probe was going down into the sample, whereas the cohesiveness [–g] was calculated while the probe was being lifted. Lower values of firmness and consistency provide better spreadability of the product on the skin and easier removal from the container [39]. A higher value of cohesiveness is responsible for faster reconstruction of its structure after hydrogel application. During textural quality analysis, it was observed that hydrogel formulations without POS (P1 and P2) and containing POS (F1 and F2) expressed comparable firmness, consistency and cohesiveness. A statistically significant improvement in all examined parameters was observed in P3 and F3 dual-cross-linked formulations (Figure 8). Higher values of texture features of P3 and F3 formulations might be associated with an increase in viscosity of these hydrogels (Figure 7). Stronger interactions between ALG, PLL and Zn^2+^ ions (formulations P3 and F3) resulted in harder consistency, higher firmness and cohesiveness. Comparing P3 to F3 hydrogels, it was also noticed that addition of POS (formulation F3) contributed to the statistically significant reduction in firmness and consistency. This change is probably related to the relaxation of the hydrogel structure by POS [40]. Lower firmness and consistency make it easy to spread the product over a larger area and thereby improve the quality of antifungal treatment and increase adherence to medication. 

### 3.4. Bioadhesive Properties

The bioadhesive property is defined as the ability of a drug form to adhere to a biological surface for a longer period of time and plays a key role in dermatological preparations. Adhesion to the skin surface for an extended time enables sustained release and absorption of active pharmaceutical ingredients, which improves the effectiveness of the treatment by maintaining a therapeutic concentration of the drug at the site of application [41].

**Figure 9:**
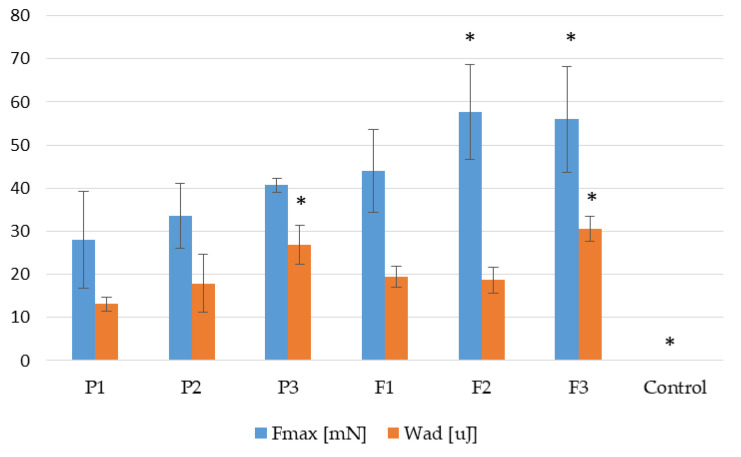
Bioadhesive properties (F_max_ and W_ad_) of dual-cross-linked hydrogels (formulations P1–P3), hydrogels containing POS (formulations F1–F3), and cellulose disc (control) (mean ± SD, *n* = 6, * significant statistical difference (*p* < 0.05)) of F_max_ and W_ad_ compared to correspondent value of P1).

To assess the performance of the developed hydrogel, placebo, and that containing POS upon interaction with the skin, bioadhesiveness tests were conducted by applying it to the skin of hairless mice to mimic in vivo conditions. The performed analysis was presented as the force of detachment (F_max_) and bioadhesiveness work (W_ad_) (Figure 9). It was demonstrated that all analyzed hydrogel formulations exhibited favourable adhesive properties. In the case of the formulation without POS, F_max_ values were obtained from 28.00 ± 11.27 mN (formulation P1) to 40.67 ± 1.53 (formulation P3) and W_ad_ from 13.13 ± 1.60 µJ (formulation P1) to 26.87 ± 4.45 µJ (formulation P3). It was concluded that the bioadhesiveness of hydrogels were dependent on the cross-linking process and the formulations composed from only ALG expressed the lowest adhesive properties.

ALG exhibits significant bioadhesive properties due to its ability to form hydrogen bonds and electrostatic interactions with epithelial cells. The interaction of alginate with biological tissues is influenced by factors such as molecular weight, the ratio of guluronic acid to mannuronic acid content, and, above all, the cross-linking process and the type and concentration of cross-linking agent used. In addition, PLL exhibits significant bioadhesive properties, primarily due to its positive charge, which enables strong electrostatic interactions with negatively charged biological surfaces such as skin, mucous membranes, and cell membranes [42]. PLL bioadhesiveness is also connected to its ability to form hydrogen bonds and ionic interactions with biological tissues, which contributes to prolonged retention of the formulation at the site of application [43]. Teng et al. constructed a series of derivatives including catechol-modified EPL (EPD), glycosylated EPL (EPG), and glucose/catechol-modified EPL (EPGD) with the same catechol content and different contents of glucose, with potential as haemostats and wound dressings, and observed their excellent adhesive properties [44]. Moreover, PLL is extensively applied as a promoter of cell adhesion [45]. Zn^2+^ ions, due to their positive charge, may also contribute to the enhancement of the bioadhesion process. This was confirmed by Dong et al., who showed that gelatin and Zn^2+^ ions present in triple-crosslinked acrylamide–*co*-acrylic acid copolymer/gelatin/Zn^2+^ hydrogels enabled excellent adhesion even during body movement [46].

The impact of a drug on hydrogel bioadhesion is not universally positive or negative—it depends on factors such as drug solubility, charge, polarity, molecular size, and compatibility with the hydrogel components. Therefore, a bioadhesiveness test for drug-loaded formulations was also evaluated. The presence of the active substance in the hydrogel matrix exhibited an increase in the magnitude of detachment force F_max_ from 44.00 ± 8.51 mN to 56.00 ± 12.29 mN. In the case of the value of W_ad_, the highest values were recorded for the F3 formulation (30.57 ± 2.80 µJ). A similar relationship was noted by Tundisi et al. Their findings demonstrated that an increase in casein concentration led to a corresponding enhancement in the bioadhesive properties of hydrogels composed of poloxamer 407 [47]. This fact may be related to the loosening of the network structure of the hydrogel as a result of the presence of the active substance and an increase in the possibilities of interaction despite the adhesive layer.

### 3.5. POS Release

Drug release from hydrogel formulations for topical application is a process that depends on many different factors, including, among others, the gel structure and composition, viscosity, water content and particle size, pH of the gel, and the medium in which the test is conducted, as well as the physicochemical properties of the formulation components [48,49]. The rate of release of the active substance from the hydrogel applied to the skin plays a key role in determining the effectiveness of local therapy of dermatological conditions. Controlled or prolonged release preparations with additional bioadhesive properties, staying for a longer time on the skin, may allow the penetration of the substance to the target skin layers while minimizing systemic absorption and local irritation [50,51].

**Figure 10:**
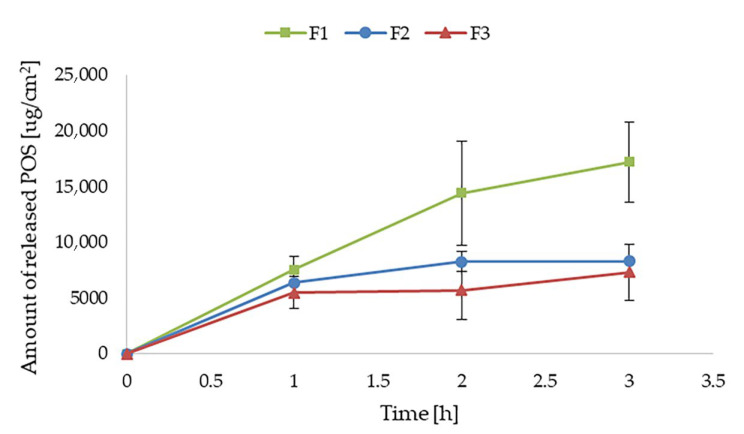
POS release from hydrogels formulations F1–F3 (mean ± SD, *n* = 3).

The release process has a meaningful impact on drug action at the skin surface and its deeper layers. The release of POS from all formulations is presented in Figure 10. It was shown that the double-cross-linking process significantly (*p* < 0.05) influenced the prolongation of the release profile of the active substance. The cumulative amount of POS released after 4 h from the hydrogels ranged from 17,224 ± 3606 μg/cm^2^ (in F1) to 7315 ± 1500 μg/cm^2^ (in F3). This phenomenon is closely associated with the formulation viscosity, which increased with the degree of cross-linking of the alginate hydrogel F1 < F2 < F3. Hydrogel viscosity plays a key role in modulating the release kinetics of incorporated drug substances [52]. The increased viscosity of the F3 formulation obtained by double cross-linking resulted in a denser hydrogel matrix, which limited the diffusion of the active substance, leading to its prolonged release. Optimization of hydrogel viscosity is therefore crucial in designing effective local drug delivery systems.

The procedure of POS release from received hydrogel formulations was investigated through the application of multiple kinetic equations (Table 3). Interestingly, the obtained data in the case of zero- and first-order kinetics showed similar linearity within the analyzed formulations. Experimental findings showed that the POS release behaviour in formulations F1 and F2 corresponded best to the Higuchi equation, evidenced by superior regression coefficients, pointing to a diffusion-driven release process. The underlying process of drug diffusion in vitro is well illustrated by the *n* diffusion exponent in the Korsmeyer–Peppas equation. In the case of formulations F2 and F3, the values of the *n* factor were within the range 0.45 < *n* < 0.89, which indicated the release of the active substance according to the non-Fickian transport. However, the *n* formulation F1 value reached above 0.89 and indicated super case type II diffusion. This transport model involves a relaxation-controlled passage of the POS drug through the disentangling swelling ALG chains, which implies that water diffusion outpaces the structural regulation of the hydrogel matrix [27,53]. It is consistent with the results obtained by Varaprasad et al., who indicated that alginate–acrylamide hydrogel obtained by the free-radical polymerization method was characterized by super case II diffusion [54].

### 3.6. Thermal Analysis Results

**Figure 11:**
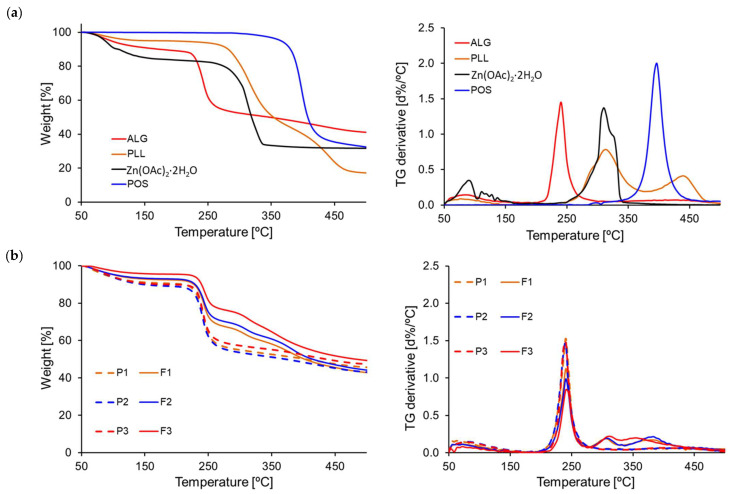
TGA and DTG curves of (**a**) hydrogel, pure components ALG, PLL, Zn(OAc)_2_·2H_2_O, and POS and (**b**) hydrogel bases without active substances (P1–P3) and containing POS (F1–F3).

To assess the properties of designed formulations under different temperature conditions, they were subjected to thermal analysis using thermogravimetric technique (TGA) and differential scanning calorimetry (DSC). TGA is a technique that measures the mass of a sample over time as the temperature fluctuates. This analysis offers insights into physical processes like phase transitions, absorption, and desorption, along with chemical processes, including thermal degradation [55]. TGA was carried out on pure formulation components—ALG, PLL, Zn^2+^, POS, and dual-cross-linked hydrogel formulations without active substance (P1, P2, P3) and formulations containing POS (F1, F2, F3) (Figure 11).

ALG TGA curves expressed three decomposition temperature steps. The first step indicative of 10.28% moisture loss was recorded at temperature at 102 °C. Remaining steps were notable at 240 °C (47.92% weight loss—the consequence of the cleavage of polymer glycosidic bonds in the course of decarboxylation and carbonization was recorded) and at 405 °C (58.95% mass reduction signalled the ALG transformation ALG into Na_2_CO_3_) [56]. Pure PLL presented adsorbed water evaporation at 90°, leading to a 5.02% weight loss. At temperatures 315 °C and 432 °C, mass losses 56.65% and 82.84% were noted, respectively. This might be associated with the breakdown of branches [9]. Similarly to ALG, zinc acetate underwent three degradation phases at 98 °C and 318 °C, resulting in a cumulative weight loss of 16.30% and 68.31%. The first step of degradation indicated the removal of water of crystallization. Conversely, POS showed only one degradation stage at 400 °C, with a mass loss of 66.78%.

The TGA analysis results of the dual-cross-linked placebo and POS-loaded hydrogels indicated that thermal decomposition initiated at higher temperatures relative to the degradation of the pure excipients. The TGA curves of all formulations showed a slight weight loss up to 150 °C, which was due to the water evaporation. Placebo formulations exhibited a three-step degradation. The first step occurred in the range of 50 °C to 180 °C. The weight loss in this range ranged from 9% for P1 formulation to 11% for P2. The second degradation step was perceived in the range of 180 °C to 280 °C, with a weight loss ranging from 32% (P3 formulations) to 35% (P1 formulation). The third step was measured between 280 °C and 500 °C. All formulations without drugs presented a comparable total weight decrease ranging from 11% (P1, P3 formulation) to 12% (P2 formulation). For formulations containing POS, an additional step in the temperature range from 330 to 340 °C to 500 °C was noted. Samples of POS-loaded hydrogels exhibited the maximum DTG peak (first derivative d%/°C) at higher temperatures compared to the empty hydrogel bases, likely associated with the decomposition of POS content at 400 °C. Based on the collected data, it can be inferred that dual-cross-linked hydrogels possessed notable thermal stability.

**Figure 12:**
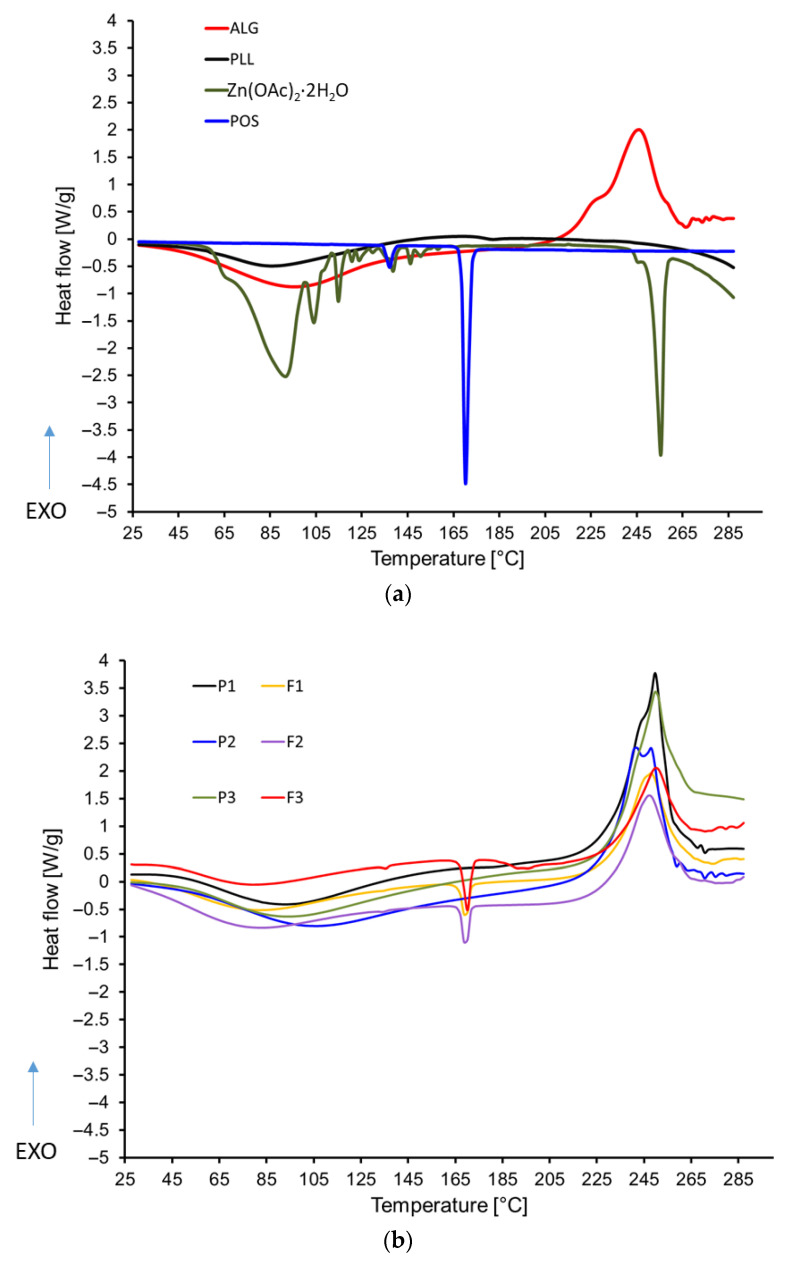
DSC thermograms of pure substances (**a**) ALG, PLL, Zn(OAc)_2_·2H_2_O, POS and (**b**) dual-cross-linked formulations without drug (P1–P3) and POS-loaded formulations (F1–F3).

Differential scanning calorimetry (DSC) is a technique widely employed in pharmaceutical analysis to gain comprehensive insights into the thermal and physical properties of active pharmaceutical ingredients (APIs) and excipients, detect potential interactions, and identify possible impurities. DSC was performed on four reference standards—ALG, PLL, Zn^2+^, POS —as well as samples without active substance (P1, P2, P3) and formulations containing POS (F1, F2, F3) (Figure 12). The analyses were conducted in an inert argon atmosphere (200 mL/min) over a temperature range of 50 to 300 °C, with a heating rate of 10 °C/min. The following slides display the DSC curves of both standards and formulations within this temperature range, including integrated peak areas obtained via the instrument’s analytical module.

ALG expressed an endothermic peak below 150 °C, attributed to the loss of moisture or residual solvents, and an exothermic peak about 247 °C, indicating the onset of thermal decomposition. The exothermic peaks observed in ALG were attributed to degradation of the polymer backbone during dehydration, as well as to depolymerization and saccharide ring destruction—processes that might result from the partial decarboxylation of protonated carboxylic groups [57]. For PLL, only an endothermic peak below 150 °C was observed, which is consistent with the evaporation of moisture or volatile solvents [58]. Zinc acetate exhibited a distinct thermal profile, characterized by a series of jagged endothermic peaks below 150 °C. These results were reproducible over multiple analyses and correlated with mass loss observed in the TGA curves, suggesting the release of crystalline water, solvents, or other volatile impurities. In the case of POS, the DSC curve revealed two distinct endothermic peaks near 137 °C and 170 °C, which are likely associated with the melting of different crystalline forms. This interpretation is confirmed by the results from the TGA analysis, where no mass loss was observed in this range. Endothermic peaks were observed in all formulations within the temperature range up to 150 °C, while exothermic peaks appeared between 225 °C and 275 °C. The endothermic peaks are attributed to the removal of moisture or residual solvents, whereas the exothermic peaks correspond to the thermal decomposition of the samples, as confirmed by weight loss observed in the TGA curves. Furthermore, in the cases of samples F1, F2, and F3, distinct endothermic melting peaks were detected around 170 °C, indicating the POS presence.

### 3.7. Thermal Analysis and Attenuated Total Reflectance–Fourier Transform Infrared Spectroscopy Evaluation

**Figure 13:**
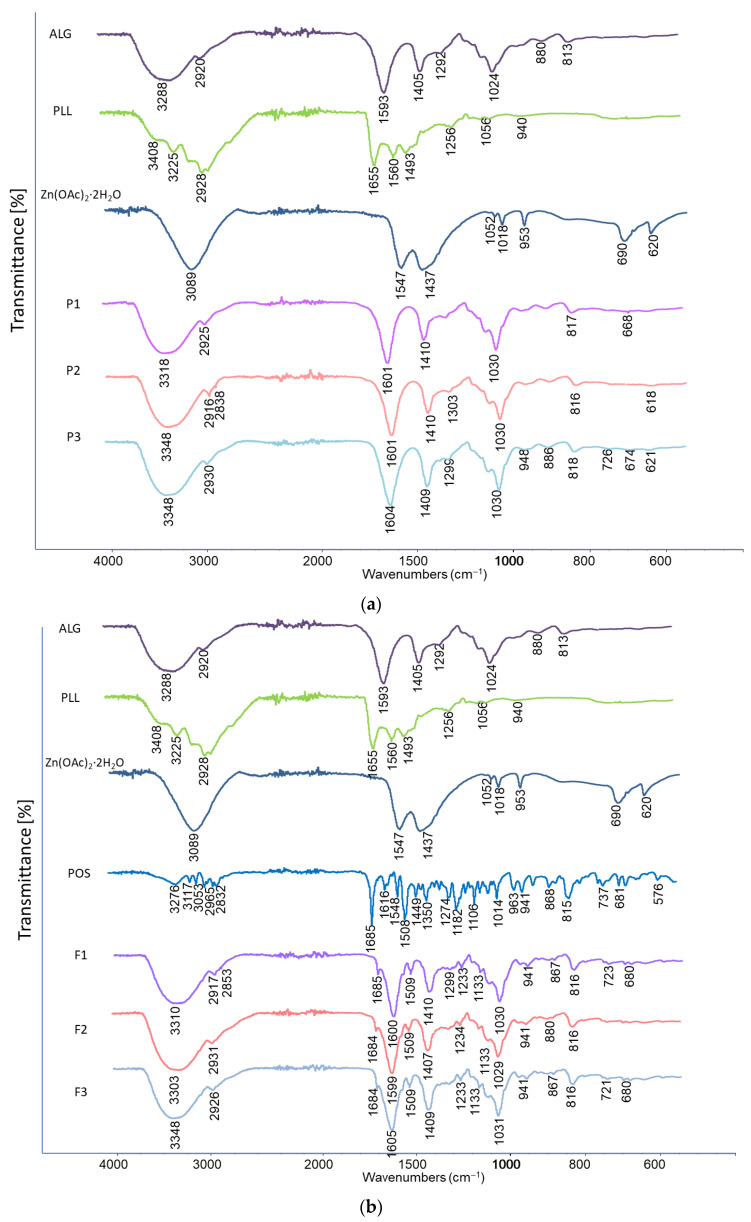
Representative FTIR-AR spectra of pure substances ALG, PLL, Zn(OAc)_2_·2H_2_O, POS and (**a**) dual-cross-linked hydrogel bases (formulation P1–P3) and (**b**) formulations containing POS (F1–F3).

Formulations with and without the drug were analyzed by FTIR (Figure 13). Spectra of the physical mixtures were dominated by signals originating from ALG. In the spectrum of pure ALG (formulation P1), a broad band corresponding to the stretching vibrations of O-H bonds was detected, with a peak at 3288 cm^−1^, along with a signal at 2920 cm^−1^ attributed to the stretching vibrations of C-H bonds. Additionally, bands associated with asymmetric (1593 cm^−1^) and symmetric (1405 cm^−1^) vibrations of the carboxylate group were observed. The spectrum also displayed stretching vibrations of C-O bonds within the pyranosyl ring at 1024 cm^−1^. In the spectra of formulation P1, all signals characteristic for ALG were noted; nevertheless, the bands which represent -O-H and C=O band vibrations were shifted towards blue light (+30 cm^−1^ and +8 cm^−1^ respectively). In the spectra of cross-linked formulations (formulation P2), and dual-cross-linked formulations (P3), no characteristic peaks for either additive were observed, probably due to their low content in the mixture being below the detection concentration (0.025% and 0.2%, respectively). Despite that, in the spectra of the formulations P2 and P3, bands of stretching vibrations of O-H originated from ALG and were blue-shifted (+30 cm^−1^ in both formulations), which may indicate the formation of polyelectrolyte complexes between PLL and ALG [59]. Furthermore, the spectrum of formulation F2 showed a slight bulge in a broad peak near 3348 cm^−1^. This indicates the presence of the amide A band, which indicates the effect of stretching and bending vibrations of the N–H bonds and stretching vibrations of the C–O and C–N double bonds of the amide group in PLL. A broader absorption band between 2500 cm^−1^ and 3590 cm^−1^ and a shift at 2916 cm^−1^ compared to P1, caused by the stretching of the NH, NH_2_, CH and CH_2_ groups, were also observed, as well as an additional peak at 1303 cm^−1^ and 2838 cm^−1^ representing the N–H vibrations originating from PLL. The peaks of COO− stretching vibrations were located at slightly higher wave numbers (1601 cm^−1^ and 1410 cm^−1^) for F2 than for sodium alginate (1593 cm^−1^ and 1405 cm^−1^), which probably indicates the interaction of the ALG COO− group with PLL counter ions [60,61]. Analysis of the double-cross-linked P3 formulation indicated the presence of zinc acetate bands, as evidenced by the presence of a peak at 948 cm^−1^ and shift to higher values in relation to P1 of the peaks in the wavelength range of 1604 cm^−1^ and 674 cm^−1^. Moreover, an increase in the band intensity in the range of 3000–3750 cm^−1^ was demonstrated. These facts indicate the occurrence of a coordination bond between ALG and Zn^2+^ ions [62].

Moreover, in the spectra corresponding to the drug formulations (F1, F2, and F3), subtle signals associated with bond vibrations characteristic of POS were detected. These include the distinctive C=O stretching vibration from the urea functional group around 1680 cm^−1^, aromatic ring stretching at 1509 cm^−1^, C-N bond stretching at 1350 cm^−1^, C-O aryl–alkyl ether vibrations at 1233 cm^−1^, C-F stretching vibrations at 1106 cm^−1^, and aromatic ring out-of-plane bending at 867 cm^−1^. The presence of these characteristic signals suggests good compatibility between the drug and the excipients.

### 3.8. Fungicidal Assessment

**Figure 14:**
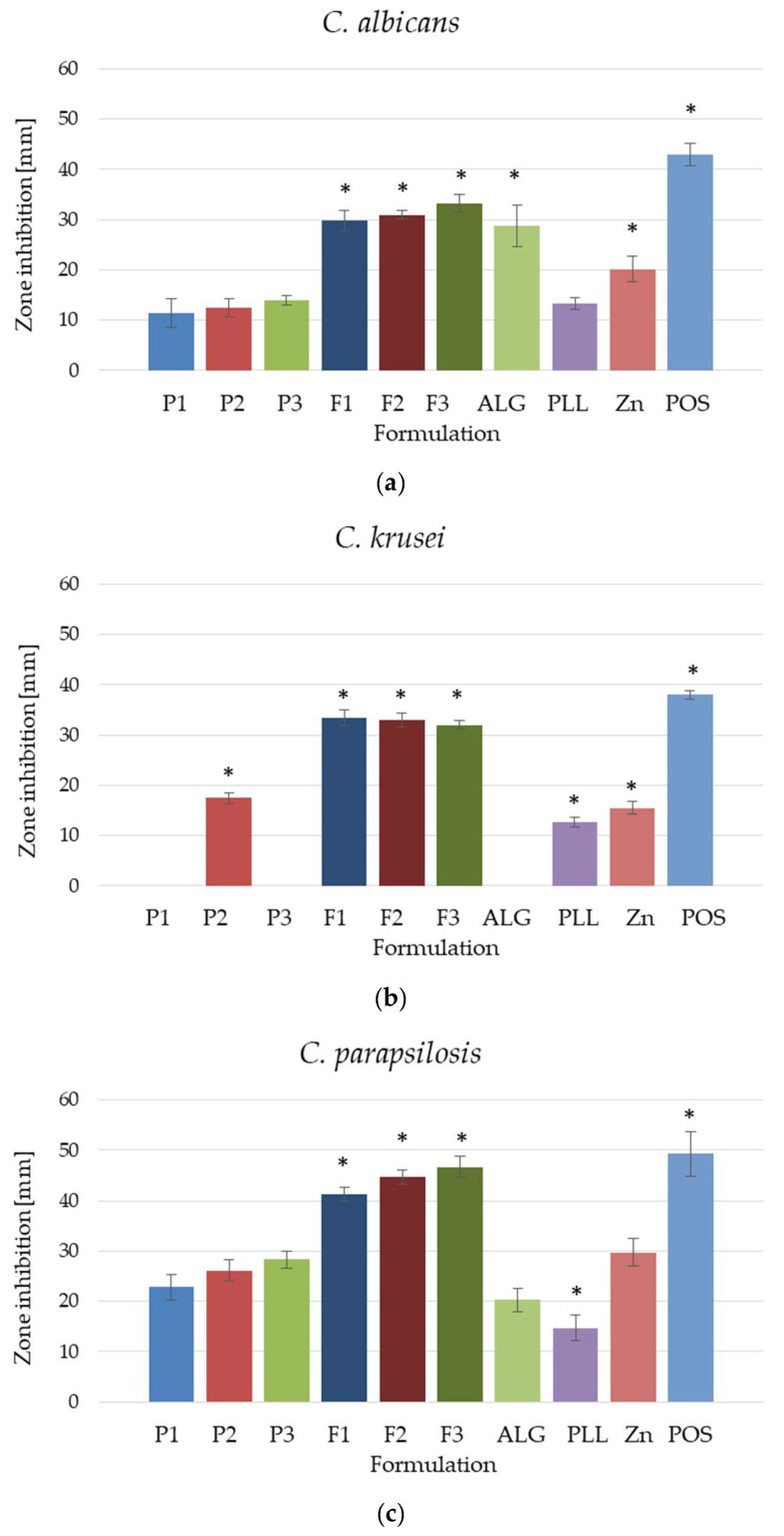
Antifungal activity of dual-cross-linked hydrogel bases (formulation P1–P3), formulations with POS (F1–F3), POS/DMSO solution, pure substances PLL, Zn(OAc)_2_·2H_2_O, and ALG for (**a**) *C. albicans*, (**b**) *C. krusei*, and (**c**) *C. parapsilosis* (mean ± SD, *n* = 6, * significant statistical difference (*p* < 0.05) versus to correspondent value of P1).

**Figure 15:**
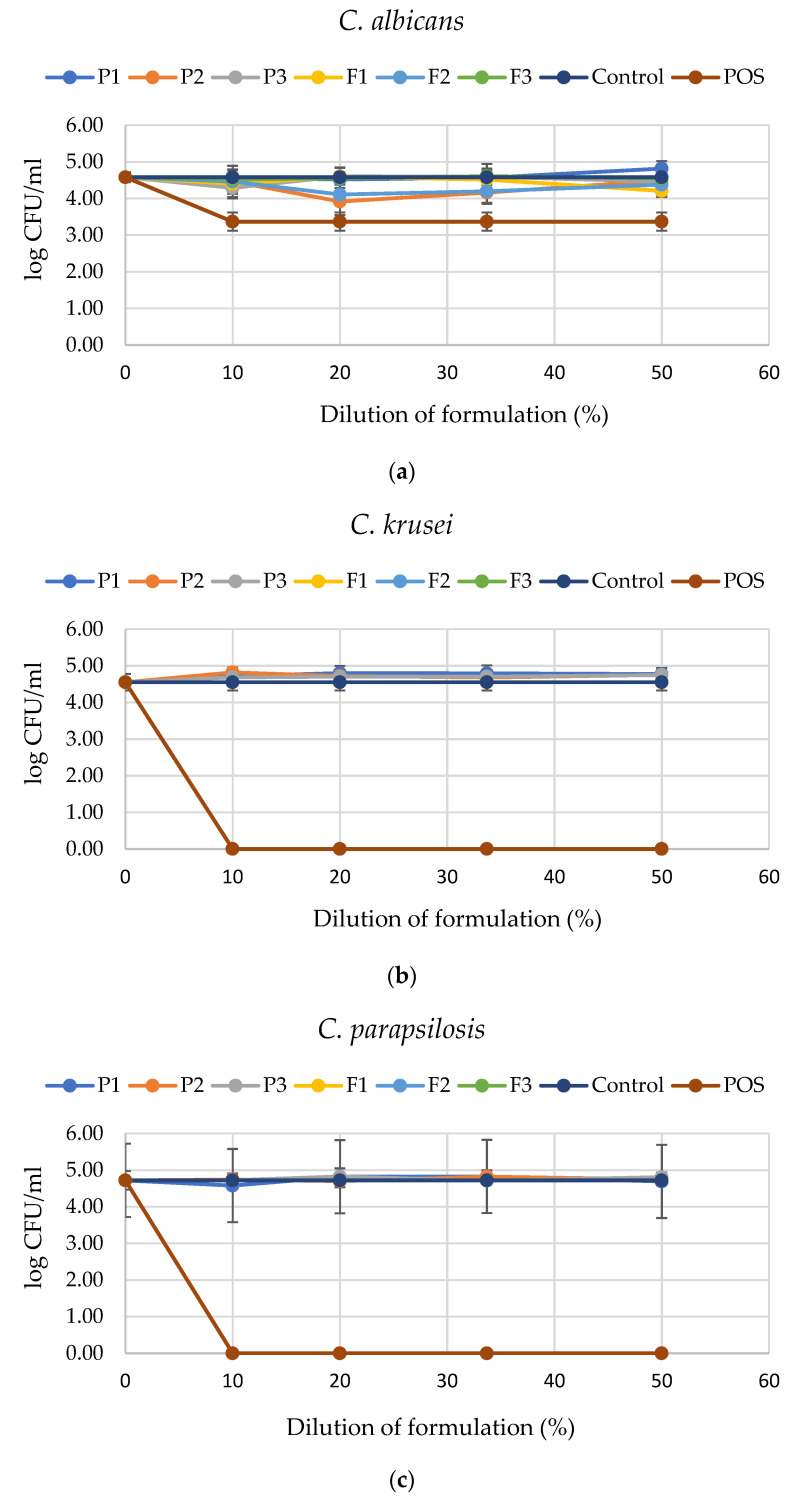
Survival of planktonic cells of (**a**) *C. albicans*, (**b**) *C. krusei*, and (**c**) *C. parapsilosis*) adjusted to the final concentration of 10, 20, 33.7, and 50% upon 1 h exposure to tested formulations without POS (P1–P3) and those containing POS (F1–F3), 1% POS in DMSO, and PBS (as control) (mean ± SD, *n* = 6).

**Figure 16:**
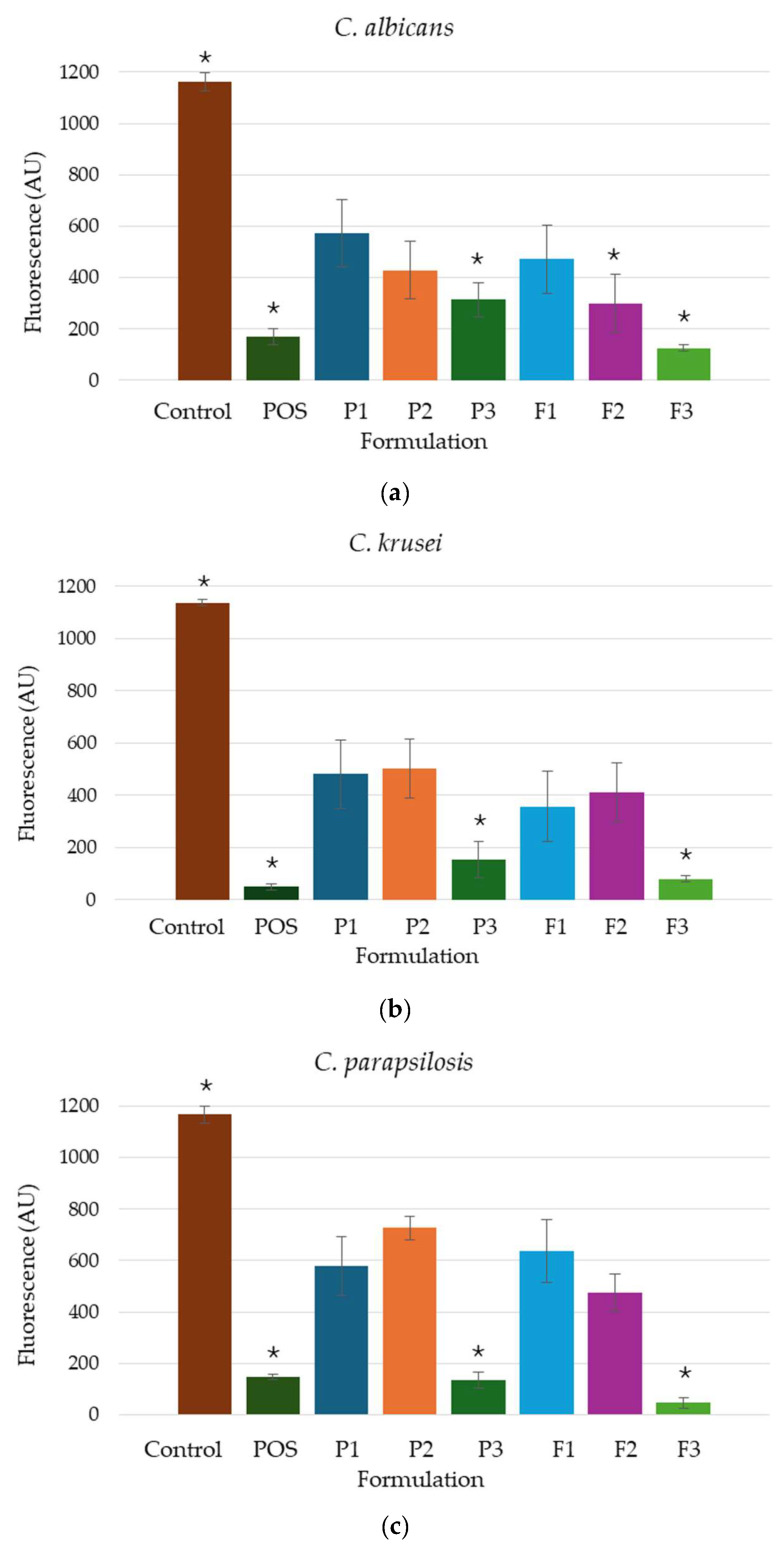
Formation of (**a**) *C. albicans*, (**b**) *C. krusei*, and (**c**) *C. parapsilosis* biofilm in the presence of tested formulations without POS (P1–P3), containing POS (F1–F3), 1% POS solution in DMSO, and PBS (as control) (mean ± SD, *n* = 6, * significant statistical difference (*p* < 0.05) versus to correspondent value of P1).

The antifungal activity of placebo hydrogel formulations and formulations with POS was assessed by the disc diffusion method (Figure 14) and against planktonic and biofilm forms (Figure 15 and Figure 16) of three different *Candida* strains: *C. albicans*, *C. krusei* and *C. parapsilosis*. The strain most sensitive to the tested formulations was *C. parapsilosis*, where the growth inhibition zone values ranged from 41.33 ± 1.53 mm for F1 to 44.86 ± 5.18 mm for F3 (Figure 14c). In the analysis of *C. albicans*, growth inhibition zones were from 29.86 ± 1.95 mm (F1) to 33.29 ± 1.80 mm (F3) (Figure 14a). In the case of both strains, dual-cross-linked formulations, both placebo and containing POS, were characterized by higher antifungal activity. The *C. krusei* strain expressed resistance to the tested formulations. Interestingly, only the P2 formulation, which was cross-linked with PLL, possessed antifungal activity among the placebo formulations, and no significant differences in growth inhibition zones were observed between the POS formulations (Figure 14b). Formulations containing POS were characterized by very similar antifungal activity at the level of 32.00 ± 0.82 (F3) to 33.43 ± 1.62 (F1).

*Candida* species are fungal pathogens responsible for numerous dermatological and systemic infections. Due to their evolving pathogenic traits and the acquisition of multiple resistance mechanisms, the treatment of candidiasis is often challenging. A distinctive characteristic of *Candida* species is their capacity to form biofilms—structured communities of microorganisms encased in a protective matrix—which enable them to evade host immune responses and resist antifungal agents. Additionally, *Candida* can develop biofilms on the skin, contributing to persistent infections and complicating treatment efforts. Accordingly, the antifungal efficacy of the formulated preparations was evaluated against both planktonic (free-floating, non-adherent) cells of *Candida* species and biofilms—structured, densely packed communities of *Candida* cells [63,64].

Experiments using planktonic *Candida* cells demonstrated that tested formulations possessed mostly limited fungicidal activity against tested strains, as indicated by the limited decrease in colony-forming unit (CFU) number detected upon overnight incubation (Figure 15). For *C. albicans*, only P2 and F2 induced some antifungal effects; nevertheless, these changes were not considerable for clinical settings and no dose-dependent effect was recorded. Similarly, the majority of formulations showed no decrease in the viability of *Candida* cells [17]. A bactericidal event was recorded for the formulation containing POS (F1–F3) and POS solutions, as their application significantly limited the viability of *C. krusei* and *C. parapsilosis* strains, even when diluted 10-fold. This indicates its high potential for the treatment of *Candida* skin diseases and proves the synergistic antifungal effect of the ingredients used in the formulation development.

The biofilm formed by *Candida* cells is resistant to many commonly active substances, which is the result of ineffective pharmacotherapy for fungal infections. Biological layer development diminishes the susceptibility of *Candida* species to antifungal agents and may contribute to the emergence of drug resistance. This reduced efficacy is attributed to the protective extracellular matrix that encases biofilm-embedded cells, hindering the penetration of antifungal compounds and limiting their access to the target cells [65]. Analysis of the developed hydrogels indicated that all formulations, both the placebo and containing POS, were characterized by antifungal activity against the analyzed strains (Figure 16). In the case of *C. albicans*, it was observed that the reduction in film formation was dependent on the presence and type of cross-linking agents. The conducted study of *C. krusei* and *C. parapsilosis* strains expressed that the presence of PLL in the formulation possessed minimal impact on the development of the microbial layer. In turn, formulation F3 possessed the most pronounced potential for surface colonization by microorganisms in the order *C. parapsilosis* > *C. krusei* > *C. albicans*. Formulations P3 and F3, containing Zn^2+^, inhibited the film formation more strongly than P1, P2, and F1, which might be caused by the sensitivity of the *Candida* spp. to zinc ions. It was shown that preparations containing zinc ions, including ZnO nanoparticles, hinder the growth of *Candida* species [66,67,68].

It is worth mentioning that the placebo formulations exhibited significant antimycotic action regardless of the test performed. This is related to the antifungal properties of ALG and cross-linking agents per se. This was demonstrated in the study of the antifungal activity of these substances in their pure form. The mode of antifungal activity exhibited by ALG remains largely unclarified. Nonetheless, various suppositions propose that ALG might generate a coating on the surface of microbial cells, thereby interfering with nutrient uptake and disrupting the normal operation of the cellular membrane. Furthermore, ALG possesses a negative charge, enabling it to engage with the outer layer of microbial organisms, potentially resulting in the efflux or seepage of intracellular substances [69]. PLL, due to its positively charged amino group, is a cationic surfactant that is well known for its strong inhibition of bacteria, fungi, and viruses. Antimicrobial activity is described by a number of different mechanisms. Scientific research proves that although the main cause of microbial death is a change in the membrane by ionic adsorption, the action is related to multidirectional synergistic damage to the microorganism. One form of damage is the inhibition of microorganism growth by affecting the integrity and permeability of their cell walls and cell membranes. As a result of the interaction of positively charged PLL groups and negatively charged phospholipid groups, the cell membrane becomes distorted and wrinkled, which leads to the formation of pores and vesicles. Increased membrane permeability causes the outflow of small molecules, K^+^ and Ca^2+^ ions, and metabolites, inducing cell death. Increased membrane permeability causes the efflux of small molecules and metabolites [8]. Additionally, PLL stimulates reactive oxygen species (ROS) production and may affect cellular responses to oxidative stress and self-defence, ultimately delaying respiration, hindering cell viability, and thus potentially inducing cell death [42]. Polycation also causes hyperpolarization of the plasma membrane potential [70]. Moreover, the second cross-linking agent—Zn^2+^ ions—exhibits notable antifungal properties, which makes it a valuable active ingredient in various dermatological formulations. Its mechanism of action involves disrupting fungal cell membrane integrity, inhibiting enzyme activity, and interfering with nutrient uptake, ultimately leading to fungal cell death. Owing to the ALG application and its double cross-linking with factors with documented antifungal properties, an enhancement in the diameter of growth inhibition zones was observed against *C. albicans* and *C. parapsilosis* strains, with increased activity towards their planktonic forms and reduced biofilm formation of all analyzed strains.

### 3.9. Biocompatibility Tests

Biocompatibility evaluation of hydrogels by applying a human fibroblast model is a key step in testing preparations intended for topical or transdermal use. Fibroblasts are metabolically active basic cells of connective tissue that synthesize and secrete components of the extracellular matrix of connective tissue. Therefore, they are a suitable model for assessing cytotoxicity, cell adhesion, and proliferation on hydrogel surfaces. Checking the hydrogel for biocompatibility with skin fibroblasts is essential to predict its safety, efficacy, and potential for integration with skin tissue [71].

**Figure 17:**
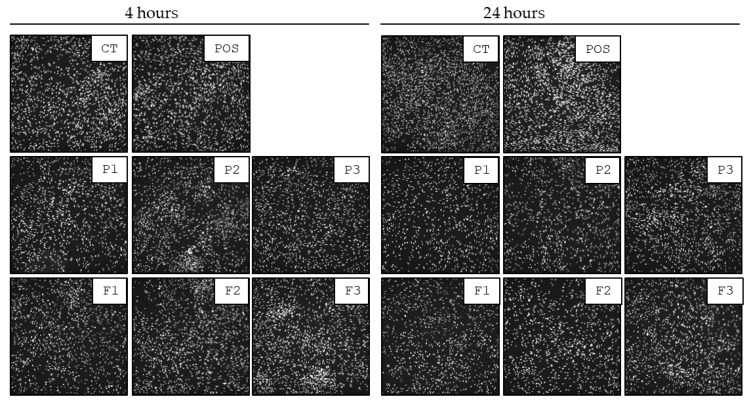
Representative microphotographs of cellular nuclei of human skin fibroblasts upon exposure to tested formulations without POS (P1–P3), containing POS (F1–F3) and untreated cells (CT) after 4 and 24 h. Scale ~500 µm.

**Figure 18:**
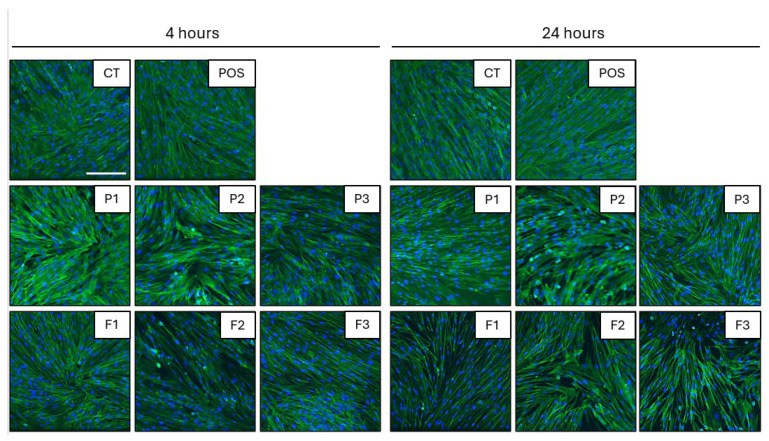
Stained cytoskeleton (green) and nuclei (blue) of human skin fibroblast cell line CCD-1070Sk (CRL-2091™) in the presence of the tested formulations without POS (P1–P3), containing POS (F1–F3) and untreated cells (CT) for 4 and 24 h. Scale ~200 µm.

**Figure 19:**
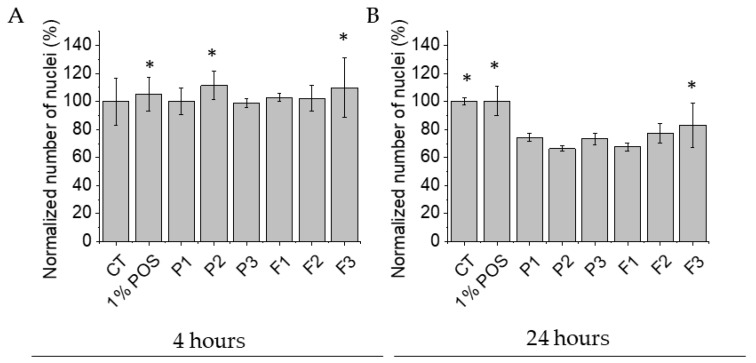
Change in the number of cell nuclei of human skin fibroblast cell line CCD-1070Sk (CRL-2091™) in the presence of tested formulations without POS (P1–P3), containing POS (F1–F3) and untreated cells (CT). Human skin fibroblast cells were left in contact with the tested formulations for 4 h (**A**) and for 24 h (**B**). The results are presented as mean ± SD and normalized to the values obtained for the control samples (*n* = 75 cells, * significant statistical difference (*p* < 0.05) versus to correspondent value of P1).

All tested formulations display biocompatibility against human skin fibroblasts. The preservation of morphology of human skin fibroblasts and the lack of significant alterations in the cell number were selected as the main determinants of the cellular biocompatibility of tested formulations. As demonstrated in Figure 17 and Figure 18, no considerable alterations in the morphology of cells exposed to tested formulations were detected up to 24 h. The images show preserved spindle morphology and strong substrate adhesion, characteristic of healthy fibroblasts. Furthermore, the biocompatibility of the material is demonstrated by the cellular shape (Figure 17) and uniform green fluorescence, characteristic of living cells (Figure 18). Although some cell shrinkage can be observed in individual combinations, cytotoxicity against the tested cell line cannot be concluded from the images obtained. To more quantitatively explore this effect, the sum of nuclei was calculated from microphotographs collected for each condition and compared with each other. As demonstrated in Figure 17, 1% POS in EMEM did not affect cell viability even up to 24 h of incubation. Similarly, no significant decrease in cellular nuclei numbers were detected after 4 h exposure of cells to tested formulations. Performed analysis expressed that all of the formulations were characterized by a non-toxic effect on fibroblast viability. Control and hydrogel formulations maintained viability levels of approximately 100% after 4 h of testing (Figure 19A). A statistically significant drop in the cell number (up to 66.34 ± 1.92% of the initial number in formulation P2) was recorded upon 24 h incubation (Figure 19B). These results align with the viability specifications of pharmaceutical products before administration to patients, based on the primary quality criteria established by the Food and Drug Administration (FDA) and in the accordance with the International Organization for Standardization (ISO) 10993 standard [72]. These standards define a limit value of 70% survival of in vitro cultures after 24 h of contact with the tested material [10,72]. The number of cell nuclei of human skin fibroblast cells decreased after 24 h in the case following hydrogel formulation application. This was probably related to the disturbance of gas exchange and uptake of components from the medium due to the density of the formulations. It can be concluded that upon short-term exposure, all tested formulations were not toxic against human skin fibroblasts in in vitro experimental settings.

## 4. Conclusions

In the summary, experimental results indicated that the double-crosslinked ALG hydrogels were successfully formulated by combining two cross-linking techniques—ionic cross-linking with Zn^2+^ ions and electrostatic interactions with positively charged PLL. The double cross-linking process increased the viscosity of the developed ALG hydrogels, improved bioadhesive properties to hairless mice skin, and provided POS an extended release profile. Furthermore, the dual cross-linking process enhanced antifungal efficacy against *C. albicans*, *C. krusei*, and *C. parapsilosis*, which might be attributed to the documented antifungal properties of the hydrogel matrix constituents. Moreover, the formulation F3 was characterized by a significant reduction in *Candida* biofilm formation. Biocompatibility tests demonstrated that all formulations were compliant with the pharmaceutical product viability specification standards.

## Figures and Tables

**Table 1:** Dual-cross-linked hydrogel formulations without active substance (P1–P3) and with POS (F1–F3).

Formulation	ALG (g)	PLL (g)	Zn^2+^ (g)	POS (g)	Water (Up To)
P1	2.0	–	–	–	100
P2	2.0	0.025	–	–	100
P3	2.0	0.025	0.2	–	100
F1	2.0	–	–	1.0	100
F2	2.0	0.025	–	1.0	100
F3	2.0	0.025	0.2	1.0	100

**Table 2:** Characteristics of designed hydrogel, placebos (formulations P1–P3), and POS-loaded hydrogels (formulations F1–F3) (mean ± SD, *n* = 3).

Formulation	pH	Mean POS Particle Size (μm)	Drug Content (%)	Viscosity * (mPa∙s)
P1	5.04 ± 0.04	–	–	2050.55 ± 57.28
P2	5.37 ± 0.01 *	–	–	2248.99 ± 286.42
P3	6.09 ± 0.02 *	–	–	5622.47 ± 973.84 *
F1	5.15 ± 0.01 *	22.87 ± 8.13	108.00 ± 3.25	1719.81 ± 57.28
F2	5.48 ± 0.02 *	25.44 ± 9.46	106.83 ± 3.76	1620.59 ± 57.28
F3	6.20 ± 0.01 *	23.52 ± 8.12	109.62 ± 1.79	4398.75 ± 206.54 *

* significant statistical difference (*p* < 0.05) versus correspondent value of P1.

**Table 3:** Models of POS release from hydrogel formulations F1–F3.

Formulation	Zero-Order Kinetics		First-Order Kinetics		Highuchi Model		Korsmeyer–Peppas Model		
	R^2^	K	R^2^	K	R^2^	K	R^2^	K	*n*
F1	0.955	4830.700	0.90	0.41	0.97	13,362.00	0.971	0.77	0.94
F2	0.77	963.68	0.76	0.13	0.93	2726.70	0.88	0.25	0.70
F3	0.85	926.61	0.86	0.15	0.79	2436.00	0.74	0.24	0.69

## Data Availability

Data are contained within the article; raw data are available upon request.

## Abbreviations

The following abbreviations are used in this manuscript:

ALG	Sodium alginate
ATR-FTIR	Attenuated Total Reflectance–Fourier Transform Infrared Spectroscopy
CFU	Colony-forming unit
CLSI	Clinical and Laboratory Standards Institute
CT	Untreated cells
DSC	Differential scanning calorimetry analysis
DMSO	Dimethyl sulfoxide
EMEM	Eagle’s Minimum Essential Medium
FBS	Fetal bovine serum
FDA	Food and Drug Administration
FTIR	Fourier Transform Infrared Spectroscopy
GRAS	Generally Regarded as Safe
HPLC	High-Performance Liquid Chromatography
ISO	International Organization for Standardization
NTU	Nephelometric turbidity unit
PEC	Polyelectrolyte complex
PLL	ε-poly-L-lysine
POS	Posaconazole
ROS	Reactive oxygen species
SEM	Scanning Electron Microscope
TGA	Thermogravimetric analysis
Zn^2+^	Zinc acetate dihydrate
